# New histopathological terminology for well-differentiated hepatocellular lesions in unusual clinico-pathological scenarios: HCA-like and FNH-like

**DOI:** 10.1016/j.jhepr.2026.101778

**Published:** 2026-02-06

**Authors:** Christine Sempoux, Venancio AF. Alves, Johanna Arola, Charles Balabaud, Paulette Bioulac-Sage, Romano Colombari, James M. Crawford, Amar P. Dhillon, Luca di Tommaso, Linda D. Ferrell, Ryan M. Gill, Maria Guido, Bilal Hameed, Kenichi Harada, Prodromos Hytiroglou, Yasuni Nakanuma, Valérie Paradis, Pierre-Emmanuel Rautou, Neil D. Theise, Swan Thung, Wilson MS. Tsui, Dale Snover, Ashley Stueck, Arief Suriawinata, Dirk J. van Leeuwen, Alberto Quaglia, International Liver Pathology Study Group

**Affiliations:** 1 Institute of Pathology, Lausanne University Hospital and University of Lausanne, Lausanne, Switzerland; 2 Department of Pathology, University of Sao Paulo School of Medicine, Sao Paulo, Brazil; 3 Department of Pathology, Helsinki University Hospital, Diagnostic Centre, and Faculty of Medicine, University of Helsinki, Helsinki, Finland; 4 Inserm, U1053, Bordeaux University, Bordeaux, France; 5 Department of Pathology, San Bonifacio Hospital, Verona, Italy; 6 Northwell Health, Department of Pathology and Laboratory Medicine, New Hyde Park, NY, USA; 7 Department of Cellular Pathology, UCL Medical School, London, UK; 8 Pathology Unit, IRCCS Humanitas Research Hospital, and Department of Biomedical Sciences, Humanitas University, Milan, Italy; 9 Department of Pathology, University of California, San Francisco, CA, USA; 10 Department of Medicine-DIMED, Pathology Unit, University of Padova, Padova, Italy; 11 Division of Gastroenterology and Hepatology, Department of Medicine, University of California, San Francisco, San Francisco, California, USA; 12 Department of Human Pathology, Kanazawa University Graduate School of Medicine, Kanazawa, Japan; 13 Department of Pathology, Aristotle University Medical School, Thessaloniki, Greece; 14 Division of Pathology, Shizuoka Cancer Center, Shizuoka, Japan, Department of Diagnostic Pathology, Fukui Prefecture Saiseikai Hospital, Fukui, Japan; 15 AP-HP, Hôpital Beaujon, Department of Pathology and Université Paris-Cité, Inserm UMR 1149, Clichy, France; 16 AP-HP, Hôpital Beaujon, Department of Hepatology and Université Paris-Cité, Inserm UMR 1149, Clichy, France; 17 Department of Pathology, New York University School of Medicine, New York, NY, USA; 18 Department of Pathology, Icahn School of Medicine at Mount Sinai, New York, NY, USA; 19 Department of Pathology, Caritas Medical Centre, Hong Kong; 20 Department of Pathology, Fairview Southdale Hospital, Edina, MN, USA; 21 Department of Pathology, Dalhousie University, Halifax, Nova Scotia, Canada; 22 Department of Pathology and Laboratory Medicine, Dartmouth Hitchcock Medical Center, Lebanon, NH, 03756, USA; 23 Section of Gastroenterology and Hepatology, Geisel School of Medicine at Dartmouth College, Hanover, NH 03755, USA; 24 Department of Cellular Pathology, Royal Free London NHS Foundation Trust, London, UK

**Keywords:** Hepatocellular adenoma, focal nodular hyperplasia, metabolic-associated steatotic liver disease, alcohol-associated liver disease, chronic liver disease, vascular liver disorders, classification, benign, malignant

## Abstract

The lines defining Hepatocellular Adenoma (HCA) and Focal Nodular Hyperplasia (FNH) can be blurred when they occur outside their prototypical clinico-pathological contexts. A terminology going beyond HCA and FNH with comments on possible malignant potential, the status of the background liver and/or underlying unusual clinical scenario is thereby justified. Indeed, both more sophisticated contrast-enhanced characterization and the identification of specific immunohistochemical and molecular markers for well-differentiated hepatocellular lesions have enabled a greater understanding of their presentation and biology. The traditional concepts of HCA and FNH occurring in livers that are “otherwise histologically normal or near normal” has given way to understanding that these lesions can also occur in livers affected by chronic liver conditions. When this is the case, morpho-molecular overlap exists between the two entities. Hence, it is now necessary to set a new approach for HCA and FNH, considering not only their intrinsic morphologic and molecular features, but also the background liver in which they are arising, and the clinical context in which they are occurring. The dichotomous paradigm of benign vs. malignant also becomes more nuanced. To raise the diagnostic uncertainty in unusual clinico-pathological scenarios, the International Liver Pathology Study Group suggests designating these lesions as *well-differentiated hepatocellular lesions*, HCA-like (and subtype when applicable) or FNH-like. This nuanced terminology enables pathologists to highlight these outliers to the hepatobiliary multidisciplinary team that will adapt the clinical management accordingly. It also provides a framework for further collaborative studies. In conclusion, we propose a more nuanced terminology, beyond conventional HCA and FNH, to consider, alongside histological and molecular features, abnormalities in the background liver and unusual clinical scenarios.

## Graphical abstract

**Figure:**
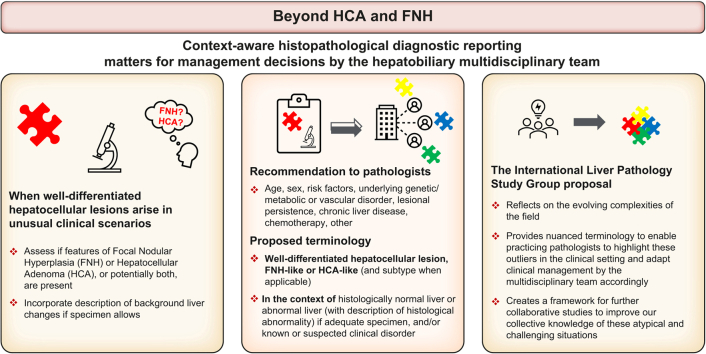

## Introduction

The last 30 years have seen a radical change in our approach to the diagnosis and classification of hepatocellular lesions in non-cirrhotic patients, brought about by close collaboration between clinicians, radiologists, pathologists and investigative scientists. This collaboration has yielded important contributions to diagnosis, such as the identification of specific immunohistochemical markers and advances in molecular biology and imaging techniques for liver tumors.

The landmark international consensus working party on the terminology of nodular hepatocellular lesions published in 1995,^1^ defined hepatocellular adenoma (HCA) as “*a benign neoplasm composed of hepatocytes occurring in the liver that is otherwise normal or nearly normal*”. The clinical setting was considered to be critical for the diagnosis of adenoma, as the vast majority of cases were thought to be due to hepatocellular stimulation related to oral contraceptives, anabolic steroids, or abnormalities in carbohydrate metabolism, such as those observed in familial diabetes mellitus, glycogen storage disease, and galactosemia. Similarly, focal nodular hyperplasia (FNH) was defined as ‘*a nodule composed of benign-appearing hepatocytes occurring in a liver that is otherwise histologically normal or nearly normal*’ supplied by large arteries, accompanied by usually prominent fibrous stroma forming a stellate scar and containing duct and ductules, representing a polyclonal proliferation of hepatocytes (*i.e.* hepatocellular hyperplasia) likely secondary to an underlying localised vascular abnormality.

## Clinico-pathological context

Decades of collaborative work have driven a progressively more granular approach to pathologic characterisation and classification of HCA. In particular, international collaborations allowed for studies powered by larger sample sizes to uncover associations between clinical, histological, and molecular features and paved the way to the identification of HCA subtypes, their associated risk factors, and clinical behaviour,[2], [3], [4] with subsequent publication of international clinical practice guidelines.^5^ The field was further advanced by recognition of novel associations between HCA and metabolic syndrome, excessive alcohol consumption, steatosis, MODY3 (maturity-onset diabetes of the young type 3) diabetes, and pathogenic alterations in *HNF1A.* Thus, these factors were considered in addition to the established contribution of sex hormones/gonadocorticoids (*i.e*. oral contraceptive and anabolic steroids) in the development of inflammatory and steatotic HCA subtypes.

**Fig. 1:**
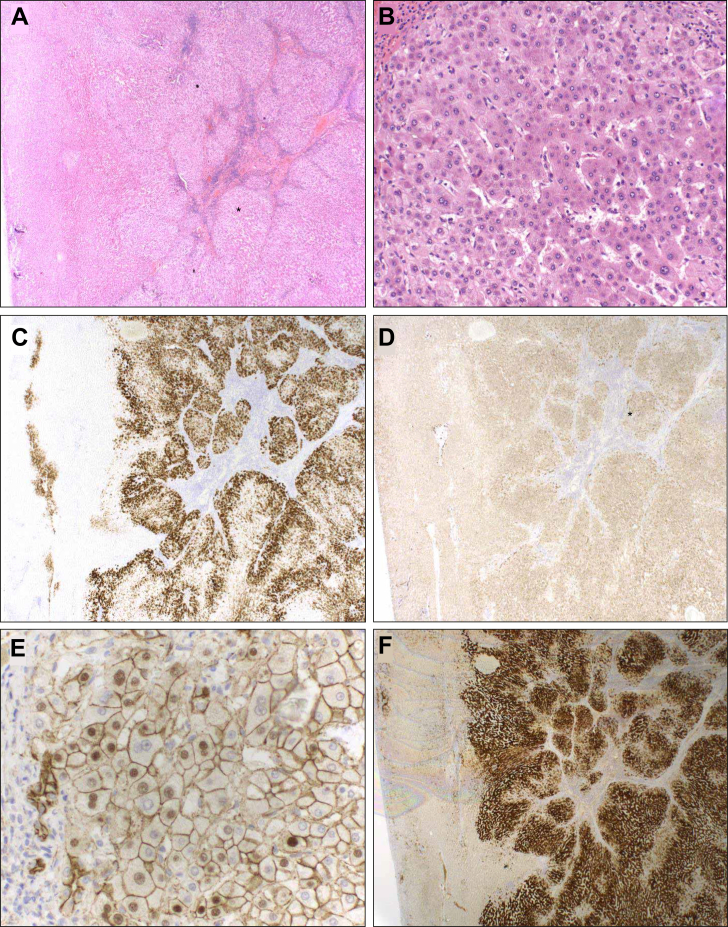
An example of a neoplastic nodule arising in the context of hepatic vascular disorders, which was traditionally thought as a background favouring the development of hyperplastic rather than neoplastic lesions. A 34-year-old man with type 2 Abernethy malformation underwent surgical resection of segment 2 and 3 for a dominant 49 mm diameter lesion. (A,B) H&E sections of the periphery of the lesion including nearby perilesional liver (left) show a nodular architecture with bridging fibrous septa and a moderate inflammatory cell infiltrate as can be observed in focal nodular hyperplasia. High magnification view in (B) of the area marked by the asterisk in (A) shows the lesional hepatic plates composed of bland hepatocytes. (C) Immunohistochemistry shows enhanced glutamine synthetase expression in a patchy pattern, resembling but not typical of a map-like pattern. (D,E) Immunohistochemistry for β-catenin shows staining of the nuclei of hepatocytes in the area marked by the asterisk in (D). (F) Immunohistochemistry for C-reactive protein shows diffuse expression. Molecular analysis revealed a c.107A>C p.(His36Pro) mutation in *CTNNB1* the gene encoding β-catenin. No *TERT* promoter mutation was identified. This case is therefore classified as a well-differentiated hepatocellular lesion, HCA-like, β-catenin mutated and inflammatory, in the setting of a type 2 Abernethy malformation.
• The high risk of malignant transformation observed in the β-catenin HCA subtype, as well as in inflammatory HCA with additional activation of the β-catenin pathway bearing certain *CTNNB1* variants, challenges the traditional view that HCAs (and, in rare cases, FNH) are benign *tout court*.^3^^,^[18], [19], [20]
• Lesions mimicking steatotic HCA in older patients can behave like HCC.^21^
• Not all HCA in men progress to HCC and even some lesions mimicking HCC on histology may follow a benign clinical course in the setting of androgen exposure and withdrawal.[22], [23], [24]
• Some HCA subtypes are prone to bleed, even if of small size.^3^^,^[25], [26], [27]
• The development of HCA in children is primarily associated with genetic syndromes, most commonly glycogen storage diseases. However, they can also occur in other predisposing conditions, such as congenital portosystemic shunts, right-sided cardiopathies, hormone exposure (*e.g.* androgen therapy for Fanconi anaemia), or MODY3. Consequently, these HCAs are often multiple and may lead to adenomatosis.^28^^,^^29^
• HNF1A HCA can be almost devoid of steatosis and become malignant, and even well-differentiated HCC may show the same pathogenic HNF1A variant.[30], [31], [32]

Many studies also showed that the lines defining these entities are often blurred, and the following situations illustrate some challenging issues:

• The inflammatory subtype of HCA was born out of a subgroup of so-called telangiectatic FNH, with somewhat atypical features for FNH, and later found to be a monoclonal lesion with molecular characteristics closer to HCA.^6^^,^^7^
• FNH and HCA, including different HCA subtypes, can occur synchronously or metachronously or even accompany hepatocellular carcinoma (HCC).^8^
• The background liver is often normal or just near normal, but can display significant changes, usually in the form of early-stage steatohepatitis and, in more extreme instances, features of advanced stage chronic liver disease, particularly alcohol-related liver disease,^9^^,^^10^ even if the majority of the nodules developing in such instances are either large regenerative nodules or dysplastic nodules.
• Lesions closely mimicking HCA and/or FNH, but often lacking all the typical features, can occur in vascular liver disorders independently of other risk factors; these lesions are more prone to malignant transformation (Fig. 1).[11], [12], [13], [14], [15], [16], [17]

In addition, histological and molecular features have helped in the development of imaging criteria for the diagnosis of liver nodules. However, standardisation and interpretation of imaging remain challenging, due to both a limited number of predictive features and overlap between them.^33^ These challenges are particularly pronounced in specific clinical settings, such as in the presence of congenital porto-systemic shunts.^34^

In contrast to classical HCA and FNH, data on lesions in these outliers or variant clinicopathological scenarios are more limited and usually based on small series or individual case reports. Despite the last 30 years of advances, the dichotomous benign *vs*. malignant approach to the interpretation of liver nodules remains deeply rooted. Designation of a hepatocellular lesion as HCA or FNH might imply that the lesion is, and will remain, indolent. However, patients can be exposed to multiple synchronous and/or metachronous risk factors, which could cumulatively contribute to neoplastic progression via a dual- or multi-hit model. A tumor may have gross and microscopic appearances considered relatively innocuous, implying that it will remain localised to its site of origin and generally amenable to local surgical removal.^35^ Yet it may still progress to malignancy over time, which would contradict the definition of ‘benign’.

Also, as in the case of progression *vs*. regression of liver fibrosis, studies of nodular lesions have focused on their static or progressive nature as defining behavioural features, rather than examining possibilities of regression. Correlative studies on regression after removal of the putative underlying stimulus (*e.g*. correction of a vascular anomaly such as closure of a porto-systemic shunt, weight loss or hormone withdrawal)^36^ are limited as regards possible histological and/or molecular ‘points of no-return’, and in terms of prediction of response or progression.

This evolution in our understanding of the scope of clinical and pathologic features in well-differentiated hepatocellular lesions argues that histological and molecular features used for the pathological diagnosis of HCA and FNH should be contextualised to the individual patient. For example, a radiologically stable lesion in a young woman taking an oral contraceptive pill, with characteristic histologic and molecular features of steatotic HCA and arising in an entirely normal liver, would match the clinico-pathological profile derived from literature on which the current classification is based and would therefore fall under the generally accepted scenario of benign behaviour and good prognosis. The only concern would be haemorrhage, the consequences in part related to its size, location and the effect of pregnancy. In contrast, an identically steatotic and bland lesion identified incidentally in the liver of a 70-year-old man would emerge as an outlier and raise concerns about its potentially malignant nature, with immediate considerations on sampling error, especially when the diagnosis relies only on a biopsy specimen. According to current histological criteria, both lesions would be classified as HCA, although the clinical approach could vary drastically depending on local expertise, protocols and patient’s preference, thus emphasising the importance of a multidisciplinary approach.

The distinction between HCA and FNH becomes particularly blurred in the context of vascular liver disorders, where these lesions are often multiple and coexisting within the same liver. Histologic evaluation has revealed overlaps in morphological, immunohistochemical and even in molecular features (Fig. 1).[11], [12], [13], [14], [15], [16], [17] These overlaps lead to a higher rate of discordance with radiological evaluation than in other situations. As may be expected, characterising these lesions by imaging alone is particularly difficult and radiologists often use the term FNH-like.^37^^,^^38^

## Proposed terminology

While the current terminology and morpho-molecular diagnostic criteria of HCA and subtypes and of FNH apply well in the prototypical contexts (*i.e.* background of clinical and/or histological normality or within the boundaries of commonly accepted risk factors and clinical scenarios), unusual clinical situations may require a different approach. We propose that when well-differentiated hepatocellular lesions reminiscent of HCA or FNH arise in unusual clinical situations, pathologists should highlight the uncertainty in biologic behaviour and lack of specific data which might be used for management decisions. We therefore recommend designating these lesions **as well-differentiated hepatocellular lesions, HCA-like (and subtype when applicable) or FNH-like,** and specifying in the report and in multidisciplinary discussions the additional information available to the pathologist which raises the diagnostic uncertainty. Known situations in which this might occur are illustrated in Box 1. This approach would not apply exclusively to biopsy specimens; the multidisciplinary discussion may also be required following histopathologic assessment of a resected lesion. Specifically, while detailed histological examination of surgically resected specimens minimises the sampling error inherent to biopsies, it may still be insufficient to categorise some liver lesions in terms of subtyping and/or malignant potential. In case of biopsy, it is recommended to complement the focused biopsies of target lesions with the documentation of background liver tissue (as through a separate needle pass of non-lesional liver), as the underlying liver disease might be unknown or require staging. Long-term follow-up can still be recommended to monitor individual patients for potential adverse outcomes, which might otherwise not be expected from the lesion’s morphology alone. Finally, this biologic uncertainty should be considered in studies correlating histological, molecular, radiological and clinical features of well-differentiated hepatocellular lesions with clinical outcomes.

Box 1 **Well-differentiated hepatocellular lesions arising in unusual clinical scenarios∗: Schematic representation for routine clinical practice.**

**Figure:**
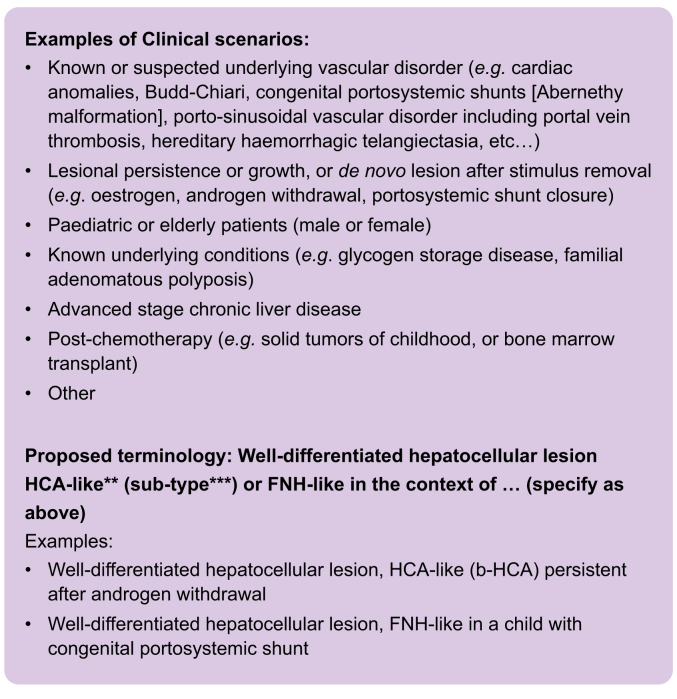

FNH, focal nodular hyperplasia; HCA, hepatocellular adenoma.

∗Unusual is intended to refer to a lesion that falls outside the boundaries of commonly accepted risk factors, age range and underlying liver abnormalities (*e.g*. oral contraceptive pill, pregnancy, *HNF1A* somatic/germline variants (MODY3), female patients of childbearing age, steatosis but not advanced fibrosis).

∗∗The hyphenated suffix ‘-like’ is our preferred terminology in this setting because it represents a simple and immediate indication of similarity to the original term (HCA or FNH). Other forms of wording (*e.g.* well-differentiated hepatocellular lesion with features of HCA or FNH) would have a similar effect and are acceptable depending on local preference. Similarly, we believe the term ‘lesion’ is globally recognised and sufficiently neutral to encompass the whole biologic spectrum. Terms such as nodule, mass or tumor, would also be acceptable, depending again on local preferences, although nodule and mass may carry a size or neoplastic connotation.

∗∗∗Subtypes as WHO Classification of tumors 5^th^ Edition, Digestive System Tumors (HNF1a-inactivated HCA (H-HCA), inflammatory HCA (IHCA), β-catenin activated HCA (b-HCA), β-catenin activated IHCA (b-IHCA), sonic hedgehog HCA (sh-HCA), unclassified.

N.B. The clinical scenarios listed above are not mutually exclusive as they might co-exist in individual patients.

Alt-text: Box 1

## Conclusion

We are thus proposing an evolution in the application of terminology describing well-differentiated hepatocellular lesions in clinico-pathologic instances of uncertainty. This recommendation is in keeping with more recent collective experience in this field.^39^^,^^40^

More collaborative studies are needed to better define these specific clinico-pathological situations. By recognising a broader biologic variability in specific scenarios for well-differentiated hepatocellular lesions, we are providing greater opportunity for management of individual patients, in support of the promise of precision medicine. In addition, we anticipate that HCA- and FNH-like entities occurring in unusual clinical settings can now be further characterised, as additional diagnostic and research modalities become available. This will also support further development of guidelines for clinical management of these lesions, across the broader settings in which they are now known to occur.

## Abbreviations

FNH, focal nodular hyperplasia; HCA, hepatocellular adenoma; HCC, hepatocellular carcinoma.

## Author’s contributions

Concept and design, drafting of the manuscript: CS and AQ.

Critical revision of the manuscript: all.

## Data availability

Not applicable.

## Financial support

The authors received no specific funding related to this work.

## Conflict of interest

Please refer to the accompanying ICMJE disclosure forms for further details.
